# The Charge of Mr. Commissioner Barlow

**Published:** 1852-04-01

**Authors:** 


### CASE OF MRS. CATHERINE CUMMING.

---

### THE CHARGE OF MR. COMMISSIONER BARLOW.

Mr. Barlow stated that the jury were directed to inquire whether Catherine Cumming, widow, now residing at 69, Queen's Road, St. John's Wood, was a lunatic, or enjoyed lucid intervals, so that she was not sufficient for the government of herself and her property; and if so, at what time, and after what manner, and how—that was the particular part of the Commission to which he wished to draw their attention, because it was upon that that they would be asked for their verdict.

The Commission also directed them to inquire into her property and her relations; but upon these two points their verdict was not required. The inquiry was limited to what they thought her present state of mind.

By the terms of the Commission, they were directed to inquire whether Mrs. Cumming was or was not an "idiot, lunatic, or of unsound mind." In law, an idiot, strictly speaking, was a person who, from the time of his birth to the present period, never enjoyed intellectual faculties. A person of unsound mind implied a person who had had the usual intellectual faculties, but from some cause had lost them. The word "lunatic," implied a person who might or might not recover his intellects at particular periods; whereas the phrase, "unsound mind," implied a continuation of the loss of faculties. But the simplest phrase was the best, and he should hereafter put it to them whether Mrs. Cumming was now a person of sound or unsound mind.

The jury would therefore have to determine, from the evidence laid before them, whether this person was now of sound mind and competent to take care of herself and her property, or whether she was of unsound mind and incapable. That was the first issue. If they came to the conclusion that she was a person of sound mind, their duty would end there; but if they came to the opposite conclusion, then there would be a second question put to them—viz., from what particular day they found she had been in that state; they would also have to observe whether, during some particular period, she had not had some lucid intervals, that she had perfectly recovered her intellectual faculties. The Commissioner then explained that the terms of the verdict must be in accordance with the principles adverted to.

The object of the inquiry was, that the protection of the law might be thrown round this lady if she was in that state in which she was alleged to be by the promoters of the Commission.

It was utterly impossible to lay down any definition of what constituted unsoundness of mind; the jury were called upon to determine whether or not in their opinion this lady was of sound or unsound mind: they would consider whether the facts proved before them led them to the conclusion that this lady was of sound or unsound mind.

The leading characteristics of an unsound mind, were prostration of the intellectual faculties on the one hand, or delusions on the other; and they were to ascertain and determine whether the different acts proved before them, and what they themselves saw in the lady herself, led to one conclusion or the other. He should have to require the unanimous opinion of at least twelve of the jury before they could find a verdict that this lady was of unsound mind. The Commissioner adverted to the presence of counsel on both sides; that was very satisfactory, inasmuch as it relieved them from much responsibility. He need not tell them, as practical men, that it very often happened that the most conclusive evidence was that which was obtained by seeing the alleged lunatic; they were entitled to see her, and to put such questions as they might think proper (suggesting that they had better be put through himself).

The Commissioner stated for the information of counsel, that when there was no counsel for the alleged lunatic, it was his custom to see the party before he went into court. He did not think it necessary, on the present occasion, as counsel appeared for her. He had, however, been to the house, as he had been informed by high authority that she was in a delicate state of bodily health; and it had been suggested as very desirable that she should be excited as little as possible; and it was nct quite clear that she would be able to come personally before them. He had left a message for her that she was to do as she thought fit as to coming into court. He had stated that he was quite sure some of the jury would go and see her if she did not come into court. He thought his so doing would relieve her from some anxiety.

